# Feasibility of Ductus Venosus Doppler Screening During First Trimester Ultrasound: Prospective Multicenter Study

**DOI:** 10.3390/medicina61081391

**Published:** 2025-07-31

**Authors:** Félicia Joinau-Zoulovits, Anissa Bouzidi, Françoise Etienne, Christine Levêque

**Affiliations:** 1 Department of Obstetrics and Gynecology, Saint-Denis Hospital Center, 93205 Saint-Denis, France; bouzidi.hanane84@gmail.com (A.B.);; 2 Department of Obstetrics and Gynecology, Paule de Viguier Hospital, CHU, 31300 Toulouse, France; c.leveque.chir@gmail.com

**Keywords:** ductus venosus, first-trimester screening, Herman score, feasibility study, congenital heart defects, ultrasound, nuchal translucency

## Abstract

*Background and Objectives*: Doppler abnormalities in the ductus venosus (DV) during the first trimester can serve as an early marker for the detection of congenital heart defects (CHDs), but the feasibility of systematically assessing the DV remains underexplored. This study aimed to evaluate the feasibility of performing DV assessments during routine first-trimester ultrasound screenings. *Materials and Methods*: A multicenter, prospective, and descriptive study was conducted, including singleton pregnancies undergoing routine ultrasound screening between 11 + 0 and 13 + 6 weeks of gestation. Sonographers were instructed to acquire DV Doppler images during the scan, and each image was blindly reviewed by an expert using predefined quality criteria. The images were categorized as “good”, “medium”, or “unsatisfactory”, and feasibility was defined as the proportion of “good” images. Factors associated with feasibility were analyzed, including sonographer satisfaction, the Herman score and the acquisition time. *Results*: Of the 87 patients included in this study, a suitable DV Doppler image was feasible in 58.6% of cases. The feasibility was significantly higher when the sonographer was satisfied with the image, when the Herman score exceeded seven (*p* = 0.01), and when the acquisition time was less than five minutes. A strong correlation was observed between the expert’s assessment and the sonographer’s satisfaction. However, the gestational age, maternal BMI, parity, and operator-perceived image quality were not significantly associated with feasibility. *Conclusions*: The Doppler assessment of the ductus venosus during first-trimester ultrasound screening is feasible and reproducible in routine clinical practice without significantly increasing the examination time. This suggests DV measurements to enhance the early nuchal translucency measurement to enhance the early detection of congenital heart defects during the first trimester.

## 1. Introduction

Congenital heart defects (CHDs) are the most common congenital anomalies, with a prevalence of approximately 9 per 1000 live births, and represent a major cause of perinatal morbidity and mortality [1]. The early detection of CHDs during pregnancy is crucial to improving neonatal outcomes but remains challenging, particularly during first-trimester ultrasound screening, where a detailed fetal echocardiography is not routinely performed [2,3,4].

The nuchal translucency (NT) measurement is the most widely used marker for early CHD detection; however, it shows only moderate sensitivity and specificity, particularly in chromosomally normal fetuses [5,6,7]. Additional markers, such as first-trimester ductus venosus (DV) Doppler assessments, have been proposed to improve early screening for CHDs [8,9,10,11]. An abnormal DV flow, characterized by an absent or reversed a-wave, has been associated with an increased risk of aneuploidy, major cardiac defects, and adverse pregnancy outcomes [12,13,14,15]. Specifically, in euploid fetuses with increased NT, an abnormal DV flow significantly increases the likelihood of CHDs, whereas the presence of a normal DV flow is associated with a lower risk [16,17].

A recent systematic review and meta-analysis (2023) evaluated the diagnostic performance of DV assessments for CHD detection during the first trimester and demonstrated that an abnormal DV flow has a good diagnostic accuracy in euploid fetuses (AUROC 0.81), while its performance remains poor in unselected or high-risk populations [18]. These findings suggest that DV assessments could play a valuable role in improving early CHD detection among low-risk, euploid populations, particularly when combined with NT measurements.

Despite its potential, the feasibility of implementing routine DV Doppler assessments during first-trimester ultrasound screening in everyday clinical practice remains underexplored. Therefore, we conducted a prospective multicenter observational study to evaluate the feasibility of obtaining a first-trimester DV Doppler image during routine ultrasound examinations. Secondary objectives included assessing the intra-observer agreement, identifying factors influencing the visualization feasibility, evaluating the learning curve, and examining the correlation between feasibility and the Herman score.

## 2. Materials and Methods

### 2.1. Study Design and Population

This prospective, multicenter observational study was conducted between December 2016 and February 2017 at Delafontaine General Hospital (Seine-Saint-Denis) and Paule de Viguier University Hospital (Toulouse), France. We included 87 women aged 18 years or older with singleton pregnancies undergoing first-trimester morphological ultrasound screening between 11 + 0 and 13 + 6 weeks of gestation. Gestational age was determined based on the first day of the last menstrual period, the first-trimester ultrasound, or by the date of embryo transfer for in vitro fertilization cases. Fetuses with suspected cardiac abnormalities or malformations prior to evaluation were excluded.

### 2.2. Ductus Venosus Doppler Assessment

Doppler assessment of the ductus venosus (DV) was performed in fetuses with a crown–rump length between 45 and 84 mm, following the Fetal Medicine Foundation (FMF) guidelines:

- Examination during fetal quiescence.
- Image magnification to include the fetal thorax and abdomen.
- Right ventral mid-sagittal view with color flow mapping showing the umbilical vein, DV, and heart.
- Small pulsed Doppler sample volume (0.5–1.0 mm) positioned in the aliasing area above the umbilical sinus.
- Insonation angle < 30°.
- Low filter setting (50–70 Hz).
- High sweep speed (2–3 cm/s) for waveform analysis.
- Qualitative assessment of the a-wave (positive, absent, or reversed).
- Manual tracing for DV pulsatility index (PIV) measurement.

All ultrasound examinations were performed using GE Voluson^TM^ E8 systems equipped with RAB6-D transabdominal 3D volumetric probes, routinely used in first-trimester obstetric imaging. No transvaginal imaging was used.

### 2.3. Image Acquisition and Quality Assessment

**Figure 1:**
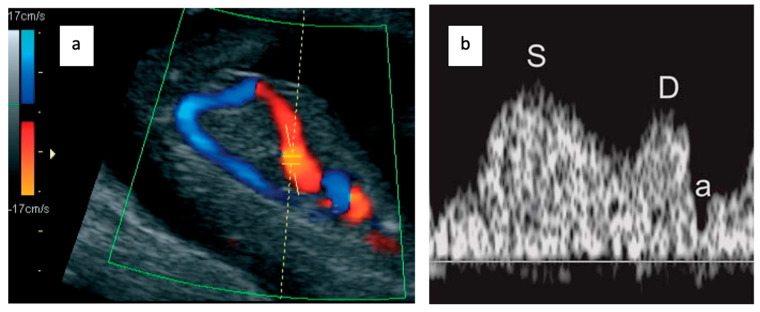
The ductus venosus color Doppler (**a**) and ductus venosus waveforms (**b**) showing the three phases: (S) systole, (D) diastole, and (a) atrial contraction.

Three trained sonographers, each aiming to recruit approximately 30 patients, performed the examinations after standardized training and validation by an expert (F.E.). For each patient, the sonographer acquired a 2D and Doppler DV image (Figure 1), selecting one representative image for expert review. The expert categorized images as “good” (all acquisition criteria met: proper sagittal view, appropriate magnification, aliasing zone targeted, angle < 30°, adequate waveform), “medium” (one criterion missing but waveform analyzable), or “unsatisfactory” (multiple criteria missing or uninterpretable waveform).

Additionally, a nuchal translucency (NT) measurement image was submitted for expert evaluation using the Herman score. Sonographers recorded the subjective feasibility, echogenicity (good, medium, poor), additional acquisition time, and perceived image quality immediately post-scan.

### 2.4. Data Collection

We prospectively collected the following data in a secure local database: maternal age, BMI, gestational age, fetal position, parity, history of uterine scarring, and examination date. Feasibility was defined as the proportion of images that the expert rated as “good”. Agreement was defined as images that were rated as “good” or “medium” by both the expert and the sonographer.

We evaluated the association of various factors with feasibility and agreement, including BMI, echogenicity, gestational age, fetal position, sonographer experience, and parity.

### 2.5. Ethics and Data Protection

This study was complied with French CNIL-MR004 and GDPR data protection regulations. It was registered under ID-RCB number 2025-A00952-47, and all participants were informed and did not object to the reuse of their data in this study.

### 2.6. Statistical Analysis

Data analyses were performed using R software (version 4.2.1). Categorical variables were compared using the chi-square (χ^2^) test, ANOVA, or Kruskal–Wallis tests as appropriate. Quantitative variables were categorized into ordinal variables for analysis. Variables significantly associated with feasibility or agreement in bivariate analysis (*p* < 0.05) were further analyzed using logistic regression to calculate unadjusted odds ratios (ORs).

## 3. Results

### 3.1. Patient Inclusion and Characteristics

A total of 87 patients were included between 1 December 2016 and 28 February 2017. Two sonographers each recruited 30 patients, while the third recruited 27 patients due to early transfers. Patient characteristics and operator-reported image quality assessments are summarized in Table 1.

Most patients were nulliparous (42/87, 48.3%) and were scanned after 12 weeks of gestation (65/87, 74.4%). The perceived image quality reported by sonographers was rated as “good” in 68 cases (78.2%), “medium” in 13 cases (14.9%), and “poor” in 6 cases (6.9%). The image was considered easy to obtain in 62 cases (72.3%), moderately easy in 19 cases (21.8%), and difficult in 6 cases (6.9%). The examination time was not significantly prolonged in 66 cases (75.9%), moderately prolonged in 18 cases (20.7%), and significantly prolonged in 3 cases (3.4%).

### 3.2. Feasibility of Ductus Venosus Assessment

The expert classified the images as “good” in 58.6% of cases (95% CI: 53–62%). Conversely, 38% of images were classified as “medium” and 3.4% as “unsatisfactory” (Table 2).

The feasibility was significantly higher when the sonographer reported satisfaction with the image (*p* < 0.01) and when the examination time was under five minutes (*p* < 0.01). The feasibility decreased when the sonographer reported a difficult fetal position (*p* = 0.03). No significant associations were found between the feasibility and the gestational age, BMI, parity, or operator-perceived image quality.

### 3.3. Agreement Between Expert and Sonographers

An agreement between the expert and the sonographer was observed in 73.6% of cases, with a Cohen’s kappa coefficient of 0.48 (95% CI: 0.30–0.66), indicating a moderate agreement beyond chance. The concordance was classified as “good” or “medium” in 71.3% of cases (62/87) (Table S1).

When the expert classified an image as “good”, the sonographer reported satisfaction in 90% of cases, whereas when the sonographer reported satisfaction, the expert agreed in 78% of cases. The agreement was significantly higher when the sonographer was satisfied (OR = 2.73, 95% CI: 1.11–4.83, *p* < 0.01) and when the examination time was not prolonged (OR = 2.98, 95% CI: 1.97–6.56, *p* < 0.03). No significant associations were found between the agreement and the BMI, echogenicity, fetal position, parity, or feasibility.

In isolated cases, discrepancies were observed between the expert and sonographer evaluations without identifiable causes.

**Figure 2:**
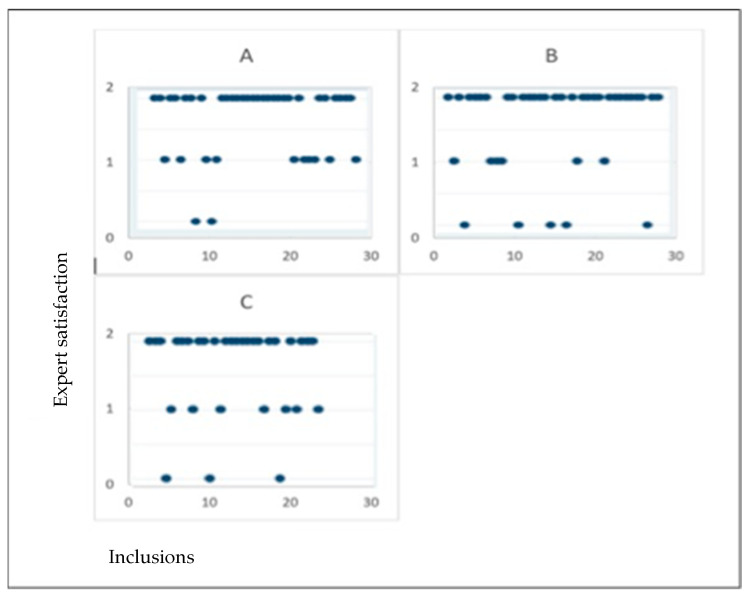
Quality of shots overtime, by sonographer quality of shots (judged by expert); 2: “good” shot, 1: “medium” shot, and 0: “unsatisfying” shot. Each dot represents a Doppler image evaluated by the expert. Image quality was rated on a 3-point scale: 2 = “good” shot, 1 = “moderate” shot, and 0 = “unsatisfactory” shot. The three panels (**A**–**C**) correspond to three different trained sonographers.

Figure 2 illustrates the evolution of image quality ratings by the sonographer over time, showing no significant differences between operators or over time, suggesting the absence of a learning curve.

### 3.4. Correlation with Herman Score

The relationship between the quality of the nuchal translucency (NT) measurement, assessed using the Herman score, and the feasibility of the DV assessment is presented in Table 3.

A Herman score > 7 was significantly associated with higher expert satisfaction (*p* = 0.01). Among cases rated as “unsatisfactory” by the expert, the Herman score was >7 in 22% and ≤7 in 78%. In contrast, among cases rated as “good,” the Herman score was >7 in 55% of cases. Feasibility was significantly higher when the Herman score exceeded seven (*p* < 0.05). When the Herman score was >7, the DV assessment was feasible according to all three sonographers in 63% of cases, likely due to the shared technical requirements for optimal NT and DV acquisition, including the sagittal plane visualization and appropriate magnification.

## 4. Discussion

### 4.1. Principal Findings

In this prospective multicenter study of 87 consecutive patients, we found that the feasibility of ductus venosus (DV) Doppler assessments during the first-trimester ultrasound reached 58.6% using a single appropriately acquired image. An agreement between the expert and sonographers was achieved in 73.6% of cases, with a higher feasibility and agreement when the sonographer reported satisfaction with the image and when the examination duration did not increase significantly. A Herman score > 7 was also associated with a higher feasibility, likely due to shared acquisition requirements between NT and DV measurements. Importantly, we observed that extending the examination time beyond 10 min often reduced the image quality and increased discrepancies between the sonographer and expert assessment, suggesting that recalling the patient may be preferable to persisting during a difficult scan.

Our findings demonstrate that a DV Doppler assessment is achievable in routine clinical practice without significantly prolonging the examination time in most cases, supporting its potential integration into first-trimester screening protocols.

### 4.2. Comparison with Previous Studies

Although our feasibility rate was 58.6%, it is important to note that Maiz et al. reported a success rate of 95% in a large-scale study specifically designed to assess the learning curve for DV Doppler acquisition [19]. Their protocol included 3000 cases performed by ten experienced sonographers, each receiving intensive and standardized training, with continuous expert supervision. In contrast, our study evaluated feasibility in routine clinical practice, without dedicated supervision and under real-world conditions. These methodological differences, including the study objectives, training intensity, and operator support, likely explain the discrepancy in feasibility rates. Our findings provide complementary insights by highlighting the practical challenges and achievable performance of DV assessments in everyday settings.

The value of the DV Doppler in first-trimester CHD screening is supported by numerous studies. An abnormal DV flow, particularly a reversed or absent a-wave, has been associated with a higher risk of CHDs, especially in euploid fetuses with increased NT [15,19,20,21,22,23]. Maiz et al., in a meta-analysis, showed that an abnormal DV flow triples the risk of CHDs when NT is ≥ the 95th percentile, while a normal DV flow halves the risk [19]. Recent large-scale data further confirmed that combining an increased NT with an abnormal DV flow enhances early CHD detection, identifying up to 47.1% of major CHDs with a 6.7% false-positive rate [24]. The underlying pathophysiology remains unclear, but associations with right-sided obstructive lesions, atrioventricular septal defects, and hypoplastic left heart syndrome suggest a link to right heart overload and diastolic dysfunction [25,26,27].

The recent 2023 meta-analysis confirmed the diagnostic accuracy of the DV Doppler for CHD detection, reporting an AUROC of 0.81 in euploid fetuses, while highlighting its lower performance in unselected populations [18]. This underscores the relevance of our study, which contributes feasibility data necessary for integrating the DV assessment in screening strategies focused on euploid pregnancies, where its performance is optimal.

### 4.3. Clinical Implications

Despite advancements in cell-free DNA testing, the early detection of major fetal anomalies, including CHDs, remains a crucial component of first-trimester screening. The addition of the DV Doppler assessment could improve early CHD detection rates, which currently remain suboptimal throughout pregnancy. The 2007 French Health Authority (HAS) report considered the DV assessment time-consuming and did not support its routine use in trisomy 21 screening protocols. However, our findings suggest that the DV assessment can be incorporated into routine practice without significant time constraints, provided sonographers are appropriately trained.

The Fetal Medicine Foundation now offers certification for the DV Doppler assessment, allowing its integration into routine first-trimester ultrasound evaluations similarly to the NT measurement. This integration may enhance the early detection of major CHDs, enabling earlier counseling, referral, and management planning for affected pregnancies.

### 4.4. Limitations and Future Directions

Our study’s limitations include the relatively small sample size (*n* = 87), which inherently restricts the statistical power and the generalizability of some subgroup analyses. While the prospective multicenter design supports external validity, we acknowledge that larger cohorts are needed to strengthen the statistical inference and to more robustly identify predictors of feasibility. Based on our findings and the observed variability, future multicenter studies should aim for a substantially larger sample size to enable more robust stratified analyses and comprehensive multivariate modeling. While our cohort size is modest compared to previous large-scale research, particularly that of Maiz et al. [20]. we believe it provides relevant feasibility insights in routine, unsupervised clinical settings. This pragmatic design reflects real-world challenges and informs the initial steps toward the broader implementation of DV Doppler assessments.

There is also potential for a selection bias, as inclusion was based on the operators’ subjective judgment regarding the ease of the image acquisition and feasibility. Moreover, we did not systematically record the exact duration required for the image acquisition, which limits the precision of our assessments regarding time efficiency. Finally, the absence of a reference comparator prevents us from fully evaluating the impact of the operator experience on the feasibility and interobserver agreement.

Although our study did not identify a clear learning curve, we recognize that the relatively short duration of this study and the limited number of cases per operator may have obscured more subtle trends in the performance improvement. Future research could benefit from extended observation periods and the use of analytical methods such as a cumulative sum (CUSUM) analysis to more accurately assess operator learning trajectories over time.

Moreover, as our study focused exclusively on feasibility, we did not include the prospective follow-up of fetal outcomes. Future research should incorporate follow-up data, particularly regarding congenital heart defects, to evaluate the predictive performance and clinical utility of the DV assessment in early screening protocols.

Future studies should also systematically record the additional time required for DV assessments in order to provide more objective evidence on its feasibility and integration into routine workflows. Importantly, the feasibility rate of 58.6% observed in our study should not be interpreted as an ideal target but rather as a pragmatic baseline in routine conditions. It underscores the need for enhanced and standardized training if the DV assessment is to be reliably integrated into screening protocols.

Finally, future research should focus on multicenter studies assessing feasibility across various operator experience levels and clinical settings, ideally including outcome correlations to confirm the added value of the DV assessment in first-trimester CHD screening protocols.

## 5. Conclusions

This prospective multicenter study demonstrates that the ductus venosus (DV) Doppler assessment during first-trimester ultrasound screening is feasible in routine clinical practice, with a success rate of 58.6%, without significantly prolonging the examination time. While this feasibility rate reflects real-world conditions—without dedicated training or supervision—it underscores the need for enhanced operator training and standardized acquisition protocols to improve performance.

Our findings suggest that the DV Doppler assessment can be realistically integrated into first-trimester screening workflows, especially when combined with the nuchal translucency (NT) measurement, to enhance early congenital heart defect (CHD) detection. Further large-scale studies, including outcome-based analyses, are needed to validate this approach, refine implementation strategies, and assess its diagnostic impact across diverse clinical settings.

## Figures and Tables

**Table 1:** Characteristics of the population.

	Average (SD ^‡^)	Total	Percentage (%)
Body mass index (BMI, kg/m^2^)	25.3 (6.3)		
Normal BMI (18–24.9)		45	51.8
Overweight (25–29.9)		28	32.2
Obesity (>30)		13	14.9
Missing values		1	1.1
Gravidity			
Primigravida		42	48.3
Multigravida		44	50.6
Missing values		1	1.1
Antecedent of uterine surgery			
Cesarian		5	5.7
Curettage		11	12.6
Gestational age	12.4 (0.58)		
≤ 12WG ^†^		22	25.3
>12WG ^†^		65	74.7
Echogenicity			
Good		68	78.2
Medium		13	14.9
Poor		6	6.9
Difficulties in obtaining the shot			
Easy		62	72.3
Medium		19	21.8
Difficult		6	6.9
Additional examination time			
None		66	75.9
Medium		18	20.7
Important		3	3.4
Satisfaction of the sonographer			
No		7	1.1
Medium		21	24.1
Yes		59	67.8

^‡^ SD: standard deviation. ^†^ WG: week of gestation.

**Table 2:** Factors associated with a “good” shot for the expert.

Expert Judgement	Total	“Good”	“Medium” or “Unsatisfactory”	*p*-Value
Total	87	51 (58.6%)	36 (41.4%)	
Sonographer				0.4
A	30	18 (60%)	12 (40%)	
B	30	15 (50%)	15 (50%)	
C	27	18 (66.7%)	9 (33.3%)	
Body mass index (BMI, kg/m^2^)				0.6
Normal (18–24.9)	45	28 (62.2%)	17 (37.8%)	
Overweight (25–29.9)	28	14 (50%)	14 (50%)	
Obesity (>30)	13	8 (61.5%)	5 (38.5%)	
Gravidity				0.5
Primigravida	42	27 (64.3%)	15 (35.7%)	
Multigravida	44	23 (52.3%)	21 (47.7%)	
Gestational age				0.2
≤12WG ^†^	22	18 (81.8%)	4 (18.2%)	
>12WG ^†^	65	41 (63.1%)	24 (36.9%)	
Echogenicity				0.1
Good	68	41 (60.2%)	26 (38.8%)	
Medium	13	8 (61.5%)	5 (38.5%)	
Poor	6	1 (16.7%)	5 (83%)	
Fetal presentation				0.03
Difficult	4	0	4 (100%)	
Not mentioned	83	50 (60.2%)	32 (38.5%)	
Additional examination time				<0.01
None	66	57 (86.4%)	9 (13.6%)	
Medium	18	2 (11.1%)	16 (88.9%)	
Important	3	0	3 (100%)	
Satisfaction of the sonographer				<0.01
No	7	1 (14.3%)	6 (85.7%)	
Medium	22	4 (18.2%)	17 (77.3%)	
Yes	58	45 (77.5%)	13 (22.4%)	

^†^ WG: week of gestation.

**Table 3:** Herman correlation and feasibility.

Herman Score	≤7	>7	Total	*p*-Value
Expert				0.01
Unsatisfied	28	8	36	
Satisfied	23	28	51	
Feasibility				<0.05
“Unsatisfactory”	3	0	3	
“Medium”	19	14	33	
“Good”	19	32	51	

## Data Availability

The original data presented in this study are openly available in Zenodo at https://doi.org/10.5281/zenodo.15827393.

## Supplementary Materials

The following supporting information can be downloaded at https://www.mdpi.com/article/10.3390/medicina61081391/s1. Table S1: Comparison between expert and sonographer judgements of shots

## Abbreviations

BMI	Body Mass Index
CHDs	Congenital Heart Defects
CRL	Crown–Rump Length
DV	Ductus Venosus
FMF	Fetal Medicine Foundation
GDPR	General Data Protection Regulation
HAS	Haute Autorité de Santé
NT	Nuchal Translucency
OR	Odds Ratio
PIV	Pulsatility Index for Veins
