# Supplementary material for: Feasibility of Ductus Venosus Doppler Screening During First Trimester Ultrasound: Prospective Multicenter Study
Source: Medicina (Kaunas). 2025 Jul 31;61(8):1391. doi: 10.3390/medicina61081391 (PMC12387703; doi:10.3390/medicina61081391)
Supplement: Supplementary file 1 [file medicina-61-01391-s001.zip › medicina-3768811-supplementary.pdf]

Table S1. Comparison between expert and sonographer judgements of shots

Sonographer<br>Expert	"Good"	"Medium"	"Unsatisfactory"	Total
"Good"	46	4	1	51
"Medium"	13	16	4	33
"Unsatisfactory"	0	1	2	3
Total	59	21	7	87
